# Preeclampsia in pregnancy affecting the stemness and differentiation potency of haematopoietic stem cell of the umbilical cord blood

**DOI:** 10.1186/s12884-020-03084-7

**Published:** 2020-07-10

**Authors:** Fazlina Nordin, Mohd Razif Mohd Idris, Zaleha Abdullah Mahdy, S. Fadilah Abd Wahid

**Affiliations:** 1 Centre for Tissue Engineering and Regenerative Medicine (CTERM), Universiti Kebangsaan Malaysia Medical Centre (UKMMC), 12th Floor, Clinical Block, Jalan Yaacob Latif, 56000 Cheras, Kuala Lumpur, Malaysia; 2 Cell Therapy Centre (CTC), UKMMC, 12th Floor, Clinical Block, Jalan Yaacob Latif, 56000 Cheras, Kuala Lumpur, Malaysia; 3 Department of Obstetrics and Gynaecology, UKMMC, Clinical Block, Jalan Yaacob Latif, 56000 Cheras, Kuala Lumpur, Malaysia

**Keywords:** Umbilical cord blood (UCB), Haematopoietic stem cell (HSC), preeclampsia (PE), Total nucleated cell (TNC), Colony forming unit (CFU)

## Abstract

**Background:**

Umbilical cord blood (UCB) has been proposed as the potential source of haematopoietic stem cells (HSC) for allogeneic transplantation. However, few studies have shown that a common disease in pregnancy such as preeclampsia would affect the quality of UCB-HSC. Total nucleated cell count (TNC) is an important parameter that can be used to predict engraftment including UCB banking. Colony forming unit (CFU) assay is widely used as an indicator to predict the success of engraftment, since direct quantitative assay for HSC proliferation is unavailable. The aim of this study is to investigate the effects of preeclampsia in pregnancy on the stemness and differentiation potency of UCB-HSC.

**Methods:**

Mononuclear cells (MNC) were isolated from UCB and further enriched for CD34+ cells using immune-magnetic method followed by CFU assay. A panel of HSC markers including differentiated haematopoietic markers were used to confirm the differentiation ability of UCB-HSC by flow cytometry analysis.

**Results/ discussion:**

The HSC progenitor’s colonies from the preeclampsia group were significantly lower compared to the control. This correlates with the low UCB volume, TNC and CD34+ cells count. In addition, the UCB-enriched CD34+ population were lymphoid progenitors and capable to differentiate into natural killer cells and T-lymphocytes.

**Conclusion:**

These findings should be taken into consideration when selecting UCB from preeclamptic mothers for banking and predicting successful treatment related to UCB transplant.

## Background

Umbilical cord blood (UCB) has been widely used as an alternative to haematopoietic stem cell (HSC) in transplantation for both malignant and non-malignant diseases including metabolic diseases. Approximately 30,000 transplantations using UCB, either from a family-related donor or non-related donor, have been reported worldwide [1]. This may be due to the ability of the HSC pool within UCB to proliferate and differentiate more rapidly, being superior to HSC extracted from bone marrow and peripheral blood [2, 3]. Additionally, it is easier to collect UCB since it poses less risk to the donor (pregnant mother), has less risk of infection, is accessible, and most importantly the loose criteria for Human Leucocyte Antigen typing match with less graft versus host disease [4]. However, the success rate of HSC transplant depends on the number and engraftment of infused nucleated cell count (NCC) and CD34+ cells [4–6]. For a standard transplant procedure, approximately 1–3 × 10^7^ cell per kg of recipient weight is needed, whereby a higher dose of infused NCC and CD34+ cells will result in a shorter period of engraftment [5]. Colony forming unit (CFU) assay is extensively used as a medium to study the capability of HSC to proliferate and differentiate by their ability to form progenitor colonies in methylcellulose semisolid media [7]. In transplantation, CFU assay is a better predictor of haematopoietic engraftment [8] and an important parameter when selecting the unit of UCB for transplantation [9]. Thus, most UCB banks have a fixed policy in selecting good quality UCB for banking based on adequate NCC and CD34+ cell counts including the ability of these cells to proliferate and differentiate [10].

Studies have reported that several obstetric factors such as maternal age, gestational age, maternal blood pressure, delivery time, route of delivery, and method of UCB collection affected the number and quality of the HSC pool within UCB [11–13]. A shorter period of UCB collection from the umbilical cord including HSC processing will give a higher number of CD34+ cells [11]. Several neonatal factors such as gender and weight also influenced the number of HSC. A heavy baby will give a high number of NCC, CD34+ cells and CFU granulocyte-monocyte [14]. The gender of the baby also produces different cell ratios with a male baby giving a higher CD34+ cells but lower NCC and CFU-granulocyte monocyte [10] while a female baby with a longer pregnancy period will give higher NCC [11]. However, not many studies reported on the quality and potency of HSC from UCB related to the above mentioned obstetric and neonatal factors in a diseased pregnancy such as in gestational diabetes mellitus (GDM) and preeclampsia. Both GDM and preeclampsia are common diseases in pregnant mothers especially for those who already have a history of diabetes and hypertension in a previous pregnancy. Stallmach et al. (1998) reported reduced endothelial cells including low hepatic haematopoiesis in the foetus, which could compromise the quality and potency of progenitor cells in UCB [15]. CD34+ cell count has also been reported to be significantly lower in patients with GDM compared to patients with hypertension and control (normal pregnancy) [16]. In this study, the quality and potency of NCC, CD34+ cells, and endothelial progenitor cells from HSC UCB will be further investigated in pregnant mothers with preeclampsia. In addition, the correlation between obstetric and neonatal factors with the quality of HSC will also be analysed. We hypothesised that preeclampsia will affect the quality and potency of HSC from UCB. These findings will be useful in guiding pregnant mothers with preeclampsia in considering future UCB banking.

## Methods

### Subjects

This cross sectional study was approved by the Research Ethics Committee of Universiti Kebangsaan Malaysia (UKM). The study was conducted on 32 women with preeclampsia, and 38 healthy pregnant women (control) who were admitted for delivery at the UKM Medical Centre (January 2014 until December 2016). The diagnosis of preeclampsia followed the definition by the American College of Obstetricians and Gynaecologists [17]. The criteria for preeclampsia included blood pressure at or above 140/90 mmHg on two readings taken 4 h apart occurring after 20 weeks of gestation, and proteinuria of at least 300 mg in a 24-h urine sample. The control group consisted of pregnant women of similar age, parity and gestational age without preeclampsia or other diseases. All subjects must test negative for hepatitis B, hepatitis C, cytomegalovirus, syphilis, and human immunodeficiency virus 1–2. Known cases of haematological disorders, genetic diseases, pre-gestational diabetes mellitus, chronic hypertension, autoimmune diseases, renal or liver impairment, multiple pregnancies and foetal anomalies or infection were excluded. Additional File 1 shows a summary of the experimental design of the study.

### Umbilical cord blood (UCB) collection

After delivery of the baby, the umbilical cord was clamped and cut, and venous UCB was collected by gravity feed. The collection was performed before placental dismissal in all patients in order to preserve the sterility of the UCB. A four to eight inch length of the cord was cleaned with alcohol and betadine. A 16-gauge needle from a standard cord blood collection bag set containing 22 ml of citrate phosphate dextrose (CPD) anticoagulant was inserted into the umbilical vein and cord blood was allowed to free-flow into a 157 ml bag. The needle was removed when blood flow stopped. A similar method was applied at caesarean section. The collection method was identical for both the preeclampsia and control groups. Collections were made by a trained staff nurse. Blood samples were stored at 4 °C immediately after collection before being handled by laboratory personnel. Samples were processed within 24 h after collection.

### Isolation of mononuclear cells (MNC) and CD34+ cells selection

Density centrifugation was performed to separate the MNC from the whole UCB. Briefly, UCB was layered on Ficoll-paque and centrifuged at 2500 rpm for 20 min, followed by aspiration of the MNC layer into new tubes with PBS in 1:3 ratios. The sample was further centrifuged at 1000 rpm for 10 min. The pellet was washed with PBS and re-suspended in 300 uL of Iscove’s Modified Dulbecco’s medium (IMDM). Subsequently 1:1 ration of blocking reagent and CD34+ microbeads were added, mixed and incubated for 30 min at 2–8 °C. The microbeads-conjugated cells were separated using a magnetic column. The isolated CD34+ cells were counted using a haemocytometer.

### Colony forming unit (CFU) assay

CFU assay was performed to observe the differentiation potency of the umbilical cord blood haematopoietic stem cell (UCB-HSC) enriched CD34+ cells and to quantify the number of each differentiated haematopoietic progenitor cells. The assay was performed using Methocult™ Kit (Stem Cell Technologies, USA). Briefly, a total of 5 × 10^3^ enriched CD34+ cells were seeded in a 35 mm petri dish with differentiation media and further incubated at 37 °C for 16 days. The CFU colonies were formed between 14 and 16 days of incubation. The HSC differentiated colonies were counted under an inverted microscope. Experiments were conducted in duplicate for each sample. Additional File 2 shows an example of UCB-HSC progenitor cell differentiation morphology with the control subject as reference.

### Flow cytometry analysis of haematopoietic stem cell markers (HSC)

The expression of HSC markers was performed in three different samples for each group. The samples include UCB-MNC, CD34+ enriched cells (after microbeads selection), and differentiated haematopoietic colonies of UCB (UCB-HSC) (the enriched CD34+ cells that went through CFU assay). The HSC markers used were CD45 (RRID:AB_2732010), CD117 (RRID:AB_2043798), CD133 (RRID:AB_244339), CD90 (RRID:AB_395969), CD34 (RRID:AB_393783), CD38 (RRID:AB_1955267), whereas CD3 (RRID:AB_393632), CD16 (RRID:AB_400104) and CD19 (RRID:AB_2629838) markers were used as an indicator for differentiated and matured haematopoietic cells. Standard direct staining was performed according to the manufacturer’s protocol (BD Bioscience US). Briefly, a total of 1 × 10^6^ cells were co-stained with 20 μL of fluorochrome-conjugated antibodies for 30 min at room temperature followed by washing 3 times using sterile 1X PBS. The expression of all markers was determined using flow cytometry (FACSCalibur™). The dot plot was analysed using CellQuestPro software (Becton Dickinson, New Jersey, USA) (RRID:SCR_014489). Additional file 3 shows an example of a dot plot analysis of HSC markers in the control sample as a reference.

### Clinical data and statistical analysis

The clinical data for each subject were obtained from the patient’s medical records. The statistical analyses were performed using SPSS 25.0 software (RRID:SCR_002865). Normality test was performed using Shapiro-Wilk test. Normally distributed continuous data were analysed using independent T-test with equal variances assumed, while continuous data that were not normally distributed were analysed using Mann-Whitney U test. Furthermore, categorical data were analysed using Fisher’s Exact Test. The correlation between UCB volume and UCB parameters was calculated using Spearman’s rank-order test. Data were shown in mean ± SD and significance is denoted with a *p*-value of less than 0.05.

## Results

### Clinical data analysis

Overall, the median age of women with preeclampsia was 33.0 (29–36) years old, which was significantly higher compared to the control group (*p = 0.008*) (Table 1). The gravidity was also significantly different between the preeclampsia and control group (*p = 0.027*) (Table 1). A total of 10 subjects in the preeclampsia group were primigravida (first pregnancy). A significantly shorter gestational period was observed in the preeclampsia group compared to the control group (*p < 0.0005*) with median of 37 weeks (Table 1). In addition, findings from this study showed that preeclampsia affects the mode of delivery. Caesarean section was significantly more frequent in the preeclampsia group compared to the control group (*p = < 0.0005*) (Table 1). The preeclampsia group had an average body mass index of 26.51 before pregnancy, which was significantly higher compared to the control group (*p = < 0.0005*) (Table 1).

**Table 1:** A comparison of maternal characteristics in PE and Control groups

Parameter	PE (***n*** = 32)	Control (***n*** = 38)	***P*** Value
**Mother’s age**	33.0 (29–36)	29.0 (27–32)	**0.008§**
**Body mass index (BMI)**	26.51 ± 3.98	22.84 ± 3.61	**< 0.0005¥**
**Pregnancy duration (week)**	37.0 (36–38)	39.0 (38–39)	**< 0.0005§**
**Leucocyte count (× 10**^**9**^**/L)**	11.0 (10–12)	11.0 (9.2–12.45)	0.967§
^*^Gravidity			
**1**	10 (31.25%)	12 (31.58%)	**0.027ǂ**
**2–3**	13 (40.62%)	24 (63.16%)	
**4–6**	9 (28.12%)	2 (5.26%)	
**Systolic blood pressure (mmHg)**	151.0 (148.5–164)	119.5 (112–130)	**< 0.0005§**
**Diastolic blood pressure (mmHg)**	92.0 (90–95.75)	69.0 (62.75–74.5)	**< 0.0005§**
^*^Delivery method			
**SVD**	18 (56.25%)	36 (94.74%)	**< 0.0005ǂ**
**LUSCS**	14 (43.75%)	2 (5.26%)	

Notes: §Mann Whitney u test (Data shown in median and interquartile)

¥ Independent sample T test (Data shown in mean ± SD)

ǂ Fisher’s Exact test

*p* value is significant < 0.05

*Shown as frequency

Abbrev: *SVD* Spontaneous Vaginal Delivery, *LUSCS* Lower Uterine Segment Caesarean Section

### UCB volume and UCB parameters analyses

The correlations between UCB volume and related parameters including NCC, TNC, CD34+ cells and total CD34+ cells were analysed. Both groups showed a significant correlation between low UCB volume and reduced TNC (*Control p = < 0.0005; Preeclampsia p = < 0.0005*) and total CD34+ cells (*Control p = < 0.0005; Preeclampsia p = 0.001*) (Table 2).

**Table 2:** A correlations between UCB volume and UCB parameters

Parameter	Group	Control (***n*** = 38)	PE (***n*** = 32)
		Volume (mL)	
**NCC (×10**^**6**^**)**	*P* value	0.061	**0.039**
	*r* value	0.307	0.366
**TNC (×10**^**8**^**)**	*P* value	**< 0.0005**	**< 0.0005**
	*r* value	0.619	0.613
**CD34**^**+**^**(cell/μL)**	*P* value	**0.038**	**0.022**
	*r* value	0.338	0.403
**Total CD34**^**+**^**cells** **(×10**^**6**^**)**	*P* value	**< 0.0005**	**0.001**
	*r* value	0.554	0.549

Notes: *p* values based on statistic Spearman’s rank order statistical analysis

*p* value is significant < 0.05

Abbrev: *NCC* nucleated cell count*, TNC* total nucleated count

### The haematopoietic differentiation of CD34+ enriched cells by CFU assay

CD34+ enriched cells from both the preeclampsia and control groups were differentiated into haematopoietic colonies or lineages. In this experiment, each colony formed in each sample were identified and counted on day 14 of the CFU assay (Table 3). The data showed a significant difference for each type of progenitor cells between the preeclampsia and control groups. Four of the colonies namely *CFU-granulocyte erythrocyte monocyte megakaryocyte, CFU-granulocyte monocyte, CFU-granulocyte and CFU-megakaryocyte* were significantly reduced in the preeclampsia group compared to the control group (*p = 0.001; p = 0.006; p = < 0.0005; p = < 0.0005* respectively) (Table 3). However, the *burst forming unit erythroid* colony, which is a primitive erythroid progenitor, remains the same in both groups (Table 3).

**Table 3:** A comparisons of haematopoietic colonies of CD34+ enriched cell between PE and Control groups

Types of progenitor	PE (***n =*** 32)	Control (***n =*** 38)	***P*** value
CFU-GEMM	75.56 ± 17.67	87.45 ± 9.00	**0.001¥**
CFU-GM	60.75 ± 18.01	70.92 ± 11.90	**0.006¥**
BFU-E	70.0 (54.25–83.75)	77.0 (67.5–82.25)	0.330§
CFU-G	73.41 ± 28.15	89.21 ± 9.03	**< 0.0005¥**
CFU-M	64.5 (52.25–77)	82.0 (77–87.25)	**< 0.0005§**

Notes: §Mann Whitney u test (Data shown in median and interquartile)

¥ Independent sample T test (Data shown in mean ± SD)

*p* value is significant < 0.05

Abbrev: *CFU-GEMM* Colony Forming Unit granulocyte erythrocyte monocyte megakaryocyte, *CFU-GM* Colony Forming Unit granulocyte monocyte*, BFU-E* Burst Forming Unit Erythroid*, CFU-G* Colony Forming Unit granulocyte and *CFU-M* Colony Forming Unit megakaryocyte

### The HSC and differentiated markers expression by flow cytometry

**Fig. 1:**
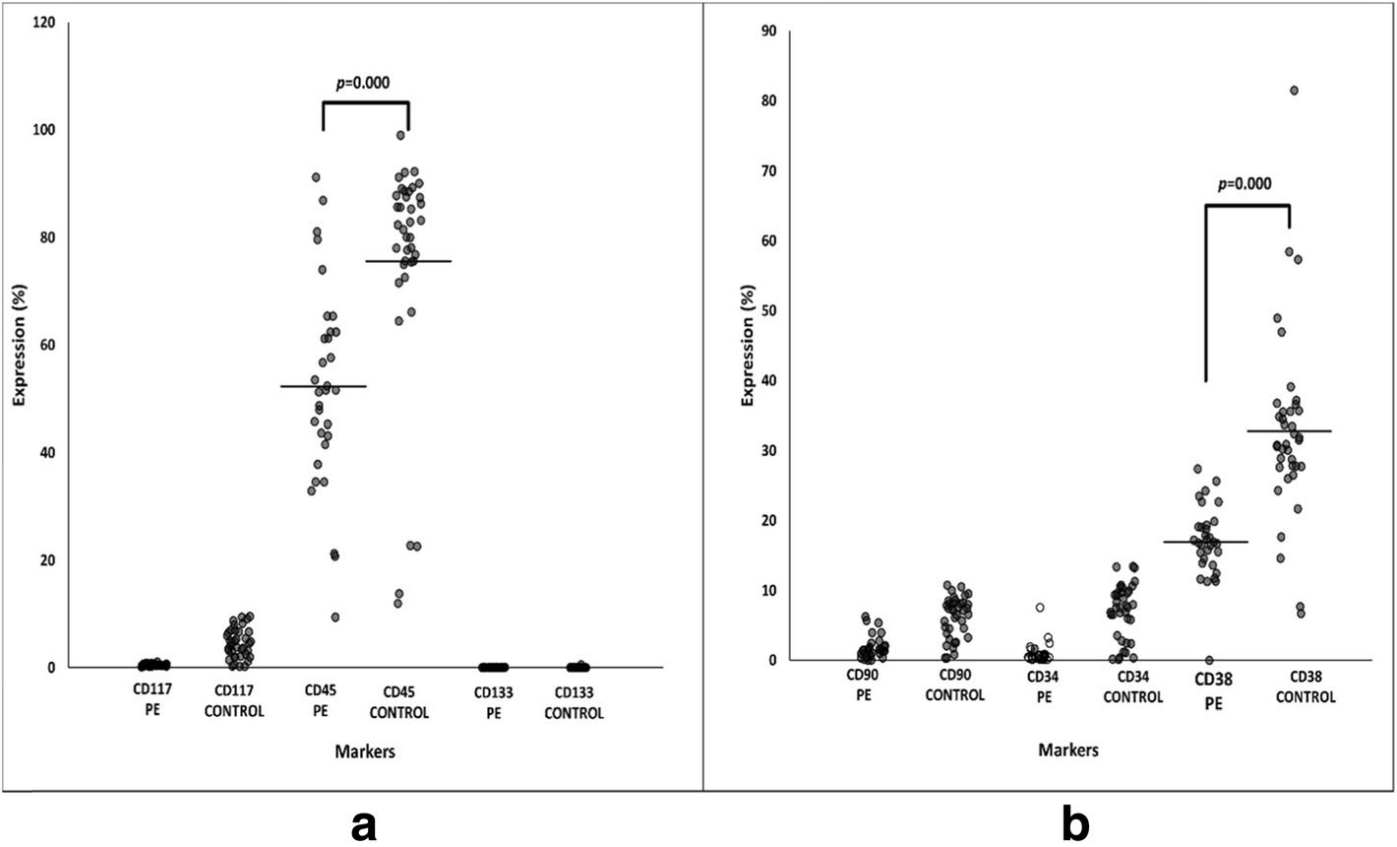
Expression of CD markers in UCB-MNC samples (pre-enriched samples). **a** CD45, CD117 and CD133 markers (**b**) CD34, CD38 and CD90 markers. (n; PE = 38 & Control = 32)

**Fig. 2:**
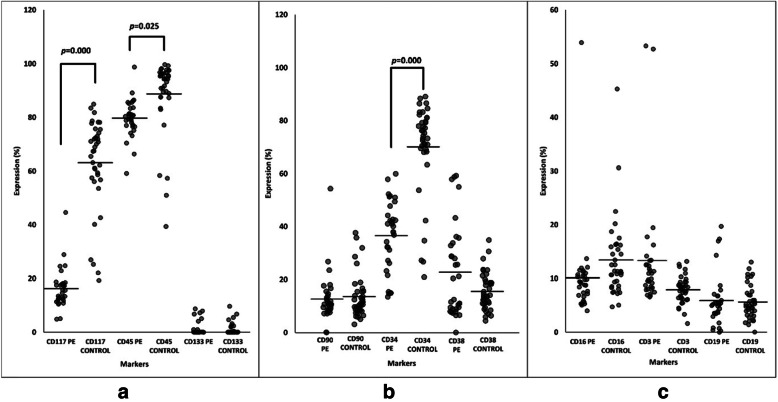
Expression of CD markers in UCB enriched CD34+ samples. **a** CD45, CD117 and CD133 markers (**b**) CD34, CD38 and CD90 markers (**c**) CD3, CD16 and CD19 markers. (n; PE = 38 & Control = 32)

**Fig. 3:**
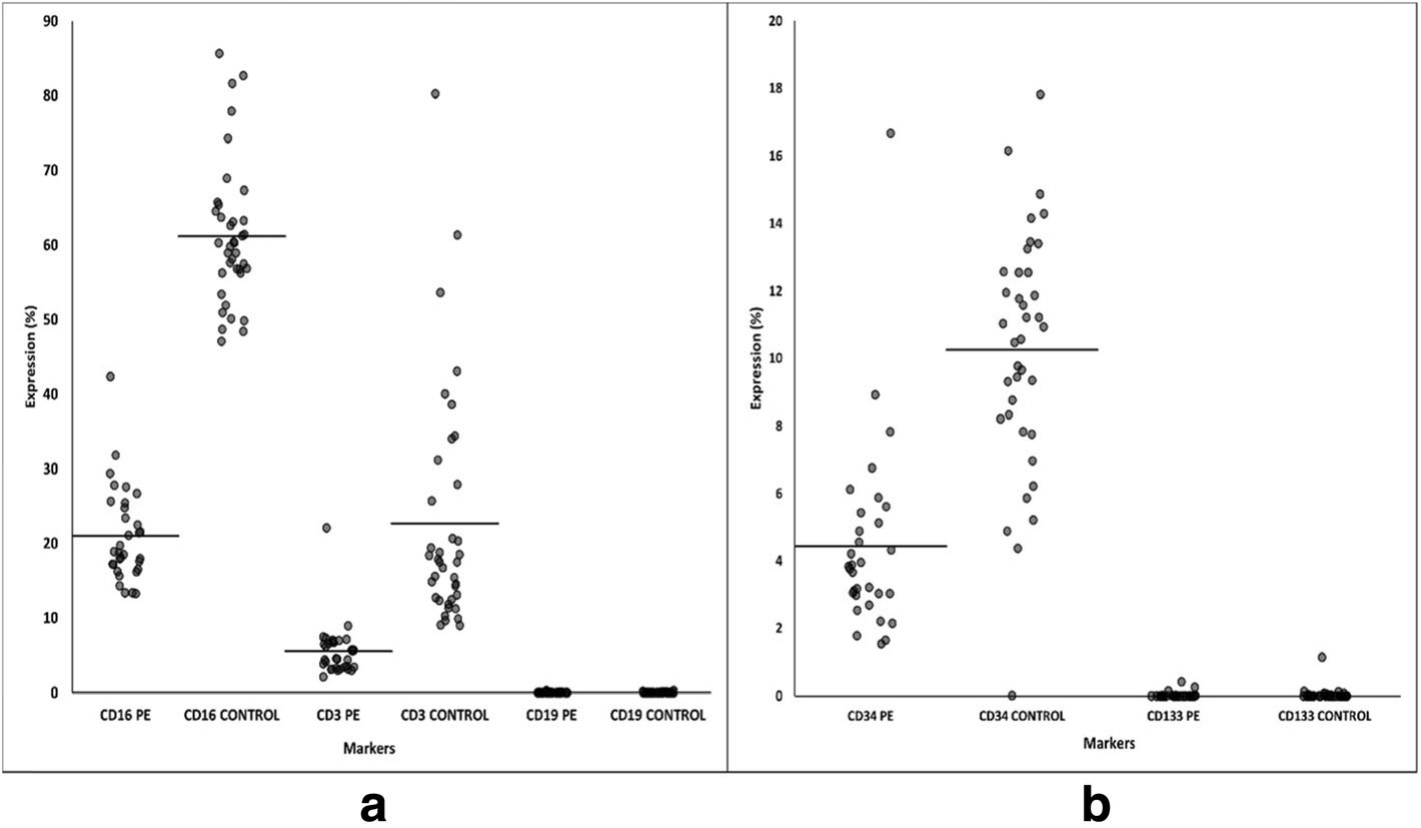
Expression of CD markers in CFU samples. **a** CD3, CD16 and CD19 markers (**b**) CD34 and CD133 markers. (n; PE = 38 & Control = 32)

The HSC markers expression in both the preeclampsia and control groups for UCB-MNC (pre-enriched samples), UCB enriched CD34+ cells (post-enriched samples) and differentiated haematopoietic cells (post-enriched and CFU assay) were compared. The UCB-MNC showed positive expression of CD45 and CD38 markers with expression over 10% in both groups (Fig. 1). The expression of CD45 and CD38 was significantly higher in the control group compared to the preeclampsia group (*p = 0.000; p = 0.000*) (Fig. 1). While in the UCB enriched CD34+ cells samples, there was an increased expression level of CD34, CD45 and CD117 markers in both groups (Fig. 2). However, the control group showed higher expression of these HSC markers compared to the preeclampsia group (Fig. 2). The *p*-values for markers of CD34+, CD45+ and CD117+ cells were *p = 0.000*, *p = 0.025* and *p = 0.000,* respectively (Fig. 2). The rest of the markers showed less than 20% increment in expression levels, which did not attain statistical significance between the two groups. HSC and selected differentiated markers expression in CFU assay for each sample were also compared. This experiment was performed to confirm that the enriched CD34+ cells were able to differentiate into haematopoietic lineages (Fig. 3). As predicted, all HSC markers expression decreased tremendously after the CFU assay. The expression of CD34 and CD133 markers decreased to less than 10% while the expression of CD16 and CD3 increased in the control group compared to the preeclampsia group (Fig. 3). However, the expression of CD19 was absent in both the preeclampsia and control groups (Fig. 3).

## Discussion

### Statement of principle findings

In a previous study performed by our group, we have confirmed that preeclampsia did affect the quality of UCB-HSC in terms of TNC and CD34+ cell counts. In this study, the investigation was extended using the CFU assay to further confirm the UCB-HSC quality in women with preeclampsia. Based on the CFU assay, the ability of UCB-HSC to differentiate into HSC progenitor colonies was shown to be affected by preeclampsia, whereby the number of colonies was significantly reduced in the preeclampsia group compared to the control group. The finding also confirmed that low UCB volume correlated with low TNC and CD34+ cells count, which may cause low numbers, hence affecting the quality of UCB-HSC differentiated cells.

### Findings in the context of existing research

#### TNC count and total CD34+ cells correlate with low UCB volume in preeclampsia compared to controls

UCB volume is the initial parameter collected at birth, thus it is an important parameter to be considered for cryopreservation and storage by most UCB banking companies worldwide. The minimum volume to qualify for UCB banking is between 40 and 60 mL for healthy mothers [18]. Our finding is in line with a previous study reported by Craig Donaldson et al. on a group of normal subjects, where he suggested that low UCB volumes are not suitable for cryopreservation [19]. Although UCB volumes collected in the preeclampsia group were more than 60 mL in this study, the TNC is significantly lower and did not adhere to the standard count of 4 × 10^8^ cells for banking purposes. A few studies have reported on the obstetric factors that affect the UCB volume, namely 1) mode of delivery (vaginal versus caesarean section), 2) induction of labour, 3) length of pregnancy, and 4) ethnicity of the mother [20]. The length of the umbilical cord may also contribute to the total UCB volume collected. Further studies are needed to determine the appropriate volume of TNC for cryopreservation.

#### A low UCB volume, low TNC and total CD34+ cells in the preeclampsia group correlates with a low number of differentiated haematopoietic colonies compared to the control group

UCB volume, TNC and total CD34+ cells are important parameters for UCB banking. Our data showed that low values of these parameters affect the low number of potential differentiated haematopoietic colonies in the CFU assay. This finding is in line with previous studies on preeclampsia [15, 21]. The higher numbers of colonies reported in the control group could be due to a good number of eminent CD34+ cells. Presumably, these eminent CD34+ cells have high potential to differentiate into HSC colonies compared to the preeclampsia group, although the same number of seeding was performed initially. CD34+ cells are a candidate for HSC progenitor marker, which will undergo in vitro haematopoiesis in this clonogenic assay [22]. The higher number of haematopoietic colonies in the control group also indicated that the CD34+ cells were able to differentiate and proliferate, suggesting that these cells were better compared to those in the preeclampsia group. This finding strengthens previous study reports on correlation between the number of CD34+ cells and TNC with the number of colonies formed [10].

### The UCB mononuclear cells (UCB-MNC) are CD45+/CD38+/CD34- suggesting a non-primitive population of HSC in both preeclampsia and control group

CD45 is a leukocyte antigen that is abundantly expressed in the MNC [23]. In the preeclampsia group, the expression of this marker was significantly reduced, which could be due to changes in the microenvironment that affect the production and composition of certain cytokines hindering the haematopoiesis process. Similar data were observed in CD38 expression whereby this marker is involved in the cell cycle that leads to the activation of CD34+ cells. This explains the low expression of CD34+ cells in UCB-MNC, where the expression level for CD34+ cells was 7% and less than 2% in the control and preeclampsia group, respectively. The CD34+ cells were less compared to the previous study with more than 14% expression level [24]. In addition, UCB-MNC showed a low expression level of CD133, CD117 and CD90 markers (< 5%). These antigens are important in stimulating the maturity of primitive CD34+ cells into functional adult HSC [23, 25]. A primitive HSC lineage is−/ CD90+/ CD45−/ CD38−/CD34+ [26]. However, contradicting data was found in this study with a lineage of−/CD45+/CD38+/CD34- in the population. According to the previous report, the primitive progenitor in UCB is highly proficient for HSC differentiation compared to other sources, making it a better candidate for cell and gene therapy [27]. Our data are consistent with results gathered from MNC of adult HSC, thus suggesting that the adult HSC exists in a dormant phase in UCB-MNC. Therefore, it can be concluded that the UCB-MNC collected in this study were a non-primitive population of HSC.

#### The UCB enriched CD34+ cells showed a significant increase of CD34, CD45 and CD117 expression in the control compared to preeclampsia group

CD117 is an antigen that is co-expressed together with CD34+ cells with a function as a marker for mature HSC. Although the expression of CD117 cells in UCB-MNC was relatively low, its expression in UCB-enriched CD34+ cells were high, up to 50 and 20% for the control and preeclampsia group, respectively. The expression level of CD117 could be varied in the CD34+ cells of the UCB sample, which could be as low as 20% or as high as 80% [28–30]. The factors that induce the differences in the expression level for CD117 are still unclear. CD34+ cells are involved in the haematopoiesis of blood cells but not in the immune system. However, the co-expression of both CD34 and CD117 markers could be interpreted as early differentiation of myeloid cells, mediated by cytokines and growth factors [31]. Although there was a slight increment of CD45 expression in the UCB-enriched CD34+ cells in the preeclampsia group, this phenomenon has never been discussed before in any study or review. It is possible that preeclampsia itself can cause changes in the microenvironment of the UCB, thus interfering with the function of the cytokines that are directly involved in the haematopoiesis process. Therefore, UCB-enriched CD34+ cells may be a primitive population compared to UCB-MNC.

#### UCB enriched CD34+ cells were lymphoid progenitors and capable to differentiate into natural killer cells and T-lymphocytes

Based on the flow cytometry analysis, it is suggested that UCB enriched CD34+ cells were lymphoid progenitors and these cells are able to differentiate into natural killer cells and T-lymphocytes. This is probably due to the nature of the cells to differentiate from an immature and primitive cell into more mature and functional cells. The CD34+ or HSC cells are progenitor cells that are pluripotent with the capability to proliferate and differentiate into multi-lineage cells [32]. By providing sufficient cytokines and growth factors, these cells are able to differentiate into *CFU-granulocyte erythrocyte monocyte megakaryocyte, CFU-granulocyte, CFU-megakaryocyte, burst forming unit erythroid and CFU-granulocyte monocyte*. In this study, a significant level of expression of CD3 and CD16 markers was observed. However, the expression of these markers was significantly higher in the control group compared to the preeclampsia group (*p* < 0.05; *p <* 0.05). CD16 expression is normally found on natural killer cells including monocytes and macrophages, while CD3 expression is a T-cell co-receptor, which is normally found in most T-cells. Interestingly, CD19 was not detected, which is normally expressed on B-cell lymphocytes. This data concluded that differentiated UCB enriched CD34+ cells were lymphoid progenitors that may further differentiate into natural killer cells and T-lymphocytes.

### Limitations and recommendation of the study

A major limitation of this study is the prospective nature and sample collection. Some suitable subjects were not consented for cord blood due to taboo beliefs. The patients and their family chose to manage all the wastes from delivery, such as cord blood and tissue, as per their own cultural beliefs or religion. Furthermore, misunderstanding on the purpose of the research, either for treatment or analysis, also results in the loss of good samples. Therefore, a presence of a counsellor or a clinician during consenting is vital to explain the purpose of the research and its outcome that will benefit other pregnant mothers with preeclampsia who are planning for cord blood banking. Another important limitation is the sample size. In this study, the sample size is considered as too small due to the cost and limit of the research grant as well as lack of resources. The small sample size might cause unreliable interpretation or false-positive results. Therefore, more preeclampsias and healthy pregnancy subjects should be included in the future study to confirm these findings. Besides that, the differences observed in UCB parameters may be partially attributable to differences in demographic and clinical characteristics between women with preeclampsia and normal pregnancies.

## Conclusion

The findings of this study suggest that CFU assay is a reliable and useful method to evaluate the quality of donor UCB from preeclampsia. We conclude that CB banking is not suitable for mothers with preeclampsia. The mothers will be advised not to banking their CB due to low volume which correlated with low TNC and CD34+ cell count.

## Supplementary information

**Additional file 1.** The diagram shows the summary of the experimental design of the present study.

**Additional file 2.** The UCB-HSC progenitors cell morphology. An example of UCB-HSC progenitor cell differentiation morphology from a control subject as reference. The scoring for each progenitor types were performed under inverted microscope after 14 days of incubation. A) CFU-GEMM, B) CFU-GM, C) BFU-E, D) CFU-G, and E) CFU-M. 4X magnifications.

**Additional file 3.** An example of dot plot analysis (flow cytometry) of HSC markers in Control sample as reference. The percentage as follows: (a) Q1 = 0.02%, Q2 = 7.18%, Q3 = 6.32%, Q4 = 86.48% (b) Q1 = 51.30%, Q2 = 3.53%, Q3 = 35.03%, Q4 = 10.14% (c) Q1 = 2.31%, Q2 = 5.31%, Q3 = 91.45%, Q4 = 0.39% (d) Q1 = 44.72%, Q2 = 10.65%, Q3 = 26.7%, Q4 = 17.85% (e) Q1 = 5.73%, Q2 = 2.22%, Q3 = 83.42%, Q4 = 8.63% (f) Q1 = 9.18%, Q2 = 0.01%, Q3 = 89.40%, Q4 = 1.41%.

## Data Availability

The datasets used and analysed during the current study are available from the corresponding author on reasonable request and after approval of the research group.

## Abbreviations

UCB: Umbilical cord blood
HSC: Hematopoietic Stem Cell
GDM: Gestational Diabetes Mellitus
TNC: Total Nucleated Cell
CFU: Colony Forming Unit
NCC: Nucleated Cell Count
MNC: Mononuclear cell
