# Supplementary material for: Preeclampsia in pregnancy affecting the stemness and differentiation potency of haematopoietic stem cell of the umbilical cord blood
Source: BMC Pregnancy Childbirth. 2020 Jul 10;20:399. doi: 10.1186/s12884-020-03084-7 (PMC7350629; doi:10.1186/s12884-020-03084-7)
Supplement: Supplementary file 1 — Additional file 1. The diagram shows the summary of the experimental design of the present study. [file 12884_2020_3084_MOESM1_ESM.pptx]

## Slide 1
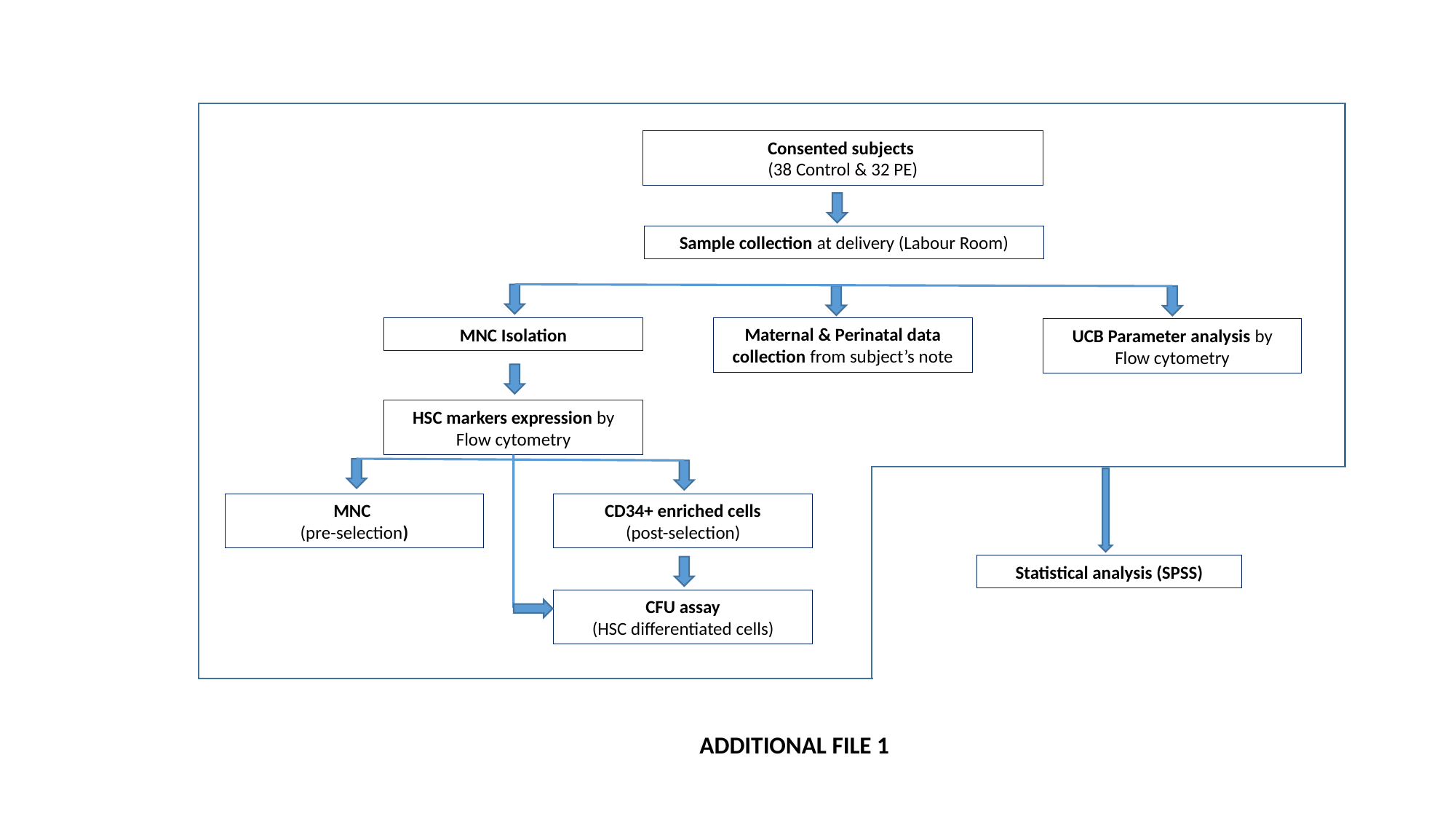

Consented subjects
(38 Control & 32 PE)
Sample collection at delivery (Labour Room)
MNC Isolation
Maternal & Perinatal data collection from subject’s note
UCB Parameter analysis by Flow cytometry
HSC markers expression by Flow cytometry
MNC
(pre-selection)
CD34+ enriched cells
(post-selection)
Statistical analysis (SPSS)
CFU assay
(HSC differentiated cells)
ADDITIONAL FILE 1
