# Supplementary figures and images for: Preeclampsia in pregnancy affecting the stemness and differentiation potency of haematopoietic stem cell of the umbilical cord blood
Source: BMC Pregnancy Childbirth. 2020 Jul 10;20:399. doi: 10.1186/s12884-020-03084-7 (PMC7350629; doi:10.1186/s12884-020-03084-7)

## Slide 1
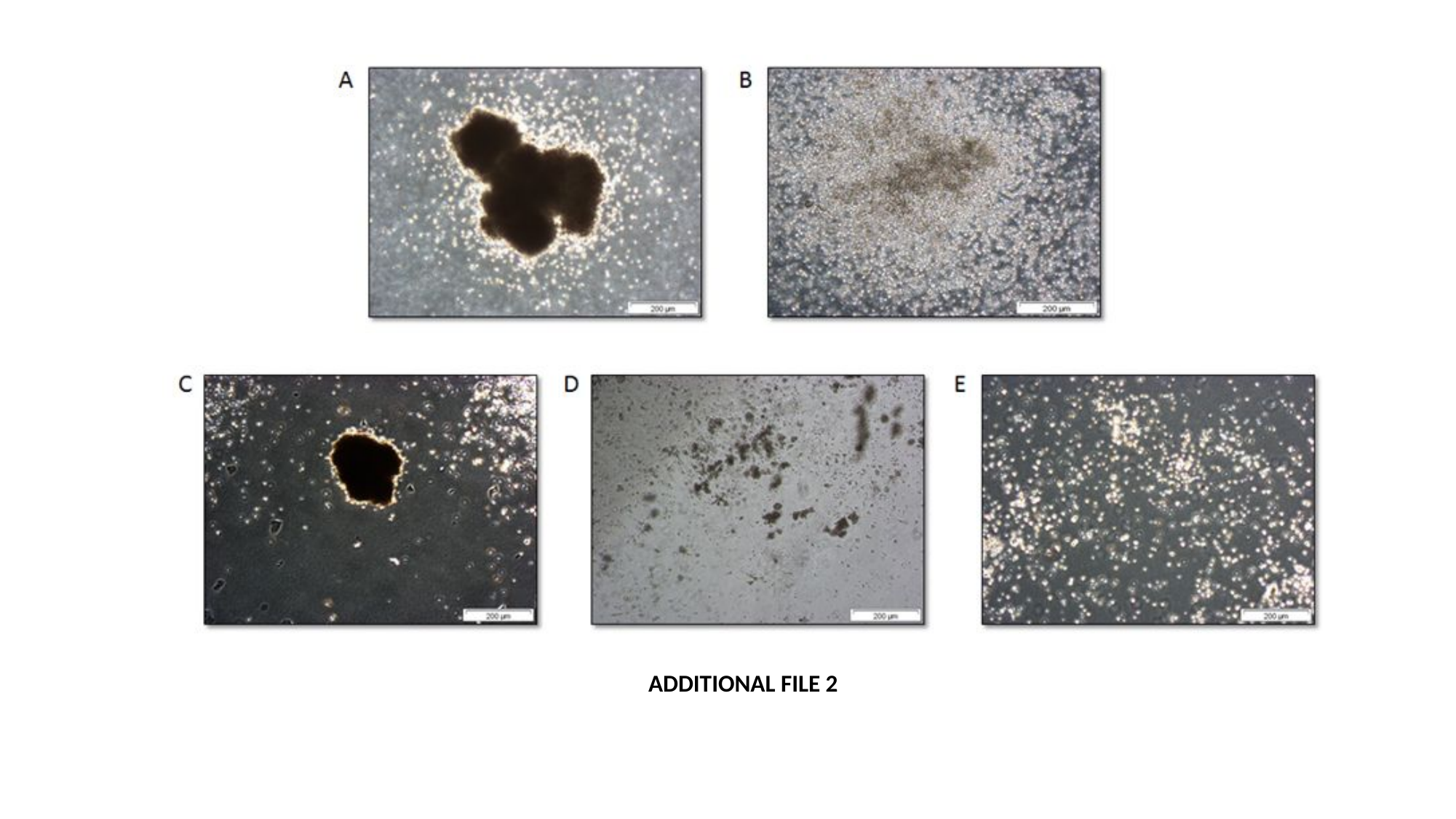

ADDITIONAL FILE 2

Supplement: Supplementary file 2 — Additional file 2. The UCB-HSC progenitors cell morphology. An example of UCB-HSC progenitor cell differentiation morphology from a control subject as reference. The scoring for each progenitor types were performed under inverted microscope after 14 days of incubation. A) CFU-GEMM, B) CFU-GM, C) BFU-E, D) CFU-G, and E) CFU-M. 4X magnifications. [file 12884_2020_3084_MOESM2_ESM.pptx]

## Slide 1
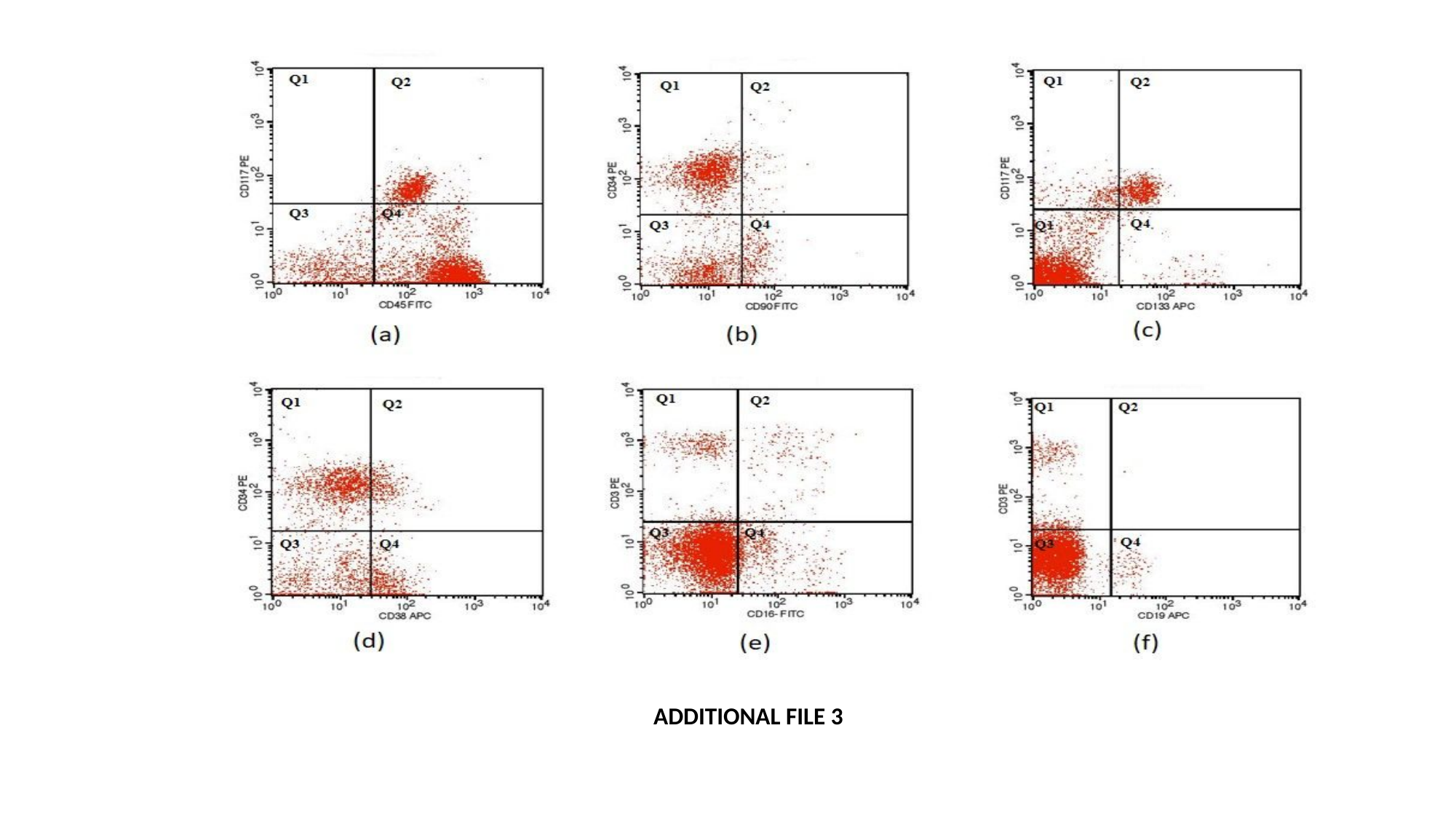

ADDITIONAL FILE 3

Supplement: Supplementary file 3 — Additional file 3. An example of dot plot analysis (flow cytometry) of HSC markers in Control sample as reference. The percentage as follows: (a) Q1 = 0.02%, Q2 = 7.18%, Q3 = 6.32%, Q4 = 86.48% (b) Q1 = 51.30%, Q2 = 3.53%, Q3 = 35.03%, Q4 = 10.14% (c) Q1 = 2.31%, Q2 = 5.31%, Q3 = 91.45%, Q4 = 0.39% (d) Q1 = 44.72%, Q2 = 10.65%, Q3 = 26.7%, Q4 = 17.85% (e) Q1 = 5.73%, Q2 = 2.22%, Q3 = 83.42%, Q4 = 8.63% (f) Q1 = 9.18%, Q2 = 0.01%, Q3 = 89.40%, Q4 = 1.41%. [file 12884_2020_3084_MOESM3_ESM.pptx]
